# Epigenetic modifications of DNA and RNA in Alzheimer’s disease

**DOI:** 10.3389/fnmol.2024.1398026

**Published:** 2024-04-25

**Authors:** Paula Martinez-Feduchi, Peng Jin, Bing Yao

**Affiliations:** Department of Human Genetics, Emory University School of Medicine, Atlanta, GA, United States

**Keywords:** Alzheimer’s disease, epigenetics, DNA methylation, non-coding RNAs, lncRNAs, miRNAs, circRNAs, RNA modifications

## Abstract

Alzheimer’s disease (AD) is a complex neurodegenerative disorder and the most common form of dementia. There are two main types of AD: familial and sporadic. Familial AD is linked to mutations in amyloid precursor protein (APP), presenilin-1 (PSEN1), and presenilin-2 (PSEN2). On the other hand, sporadic AD is the more common form of the disease and has genetic, epigenetic, and environmental components that influence disease onset and progression. Investigating the epigenetic mechanisms associated with AD is essential for increasing understanding of pathology and identifying biomarkers for diagnosis and treatment. Chemical covalent modifications on DNA and RNA can epigenetically regulate gene expression at transcriptional and post-transcriptional levels and play protective or pathological roles in AD and other neurodegenerative diseases.

## 1 Introduction

Alzheimer’s disease (AD) is a progressive neurodegenerative disorder characterized by cognitive decline and memory deficits. AD accounts for 60–80% of all dementia cases and is estimated to affect 13.8 million people in the United States by 2050 (Alzheimer’s Association, 2023). Despite the prevalence of AD, a definitive diagnosis is only possible through postmortem histological evaluation indicating the presence of amyloid-beta (Aβ) plaques, neurofibrillary tangles (NFTs), and neuronal atrophy (Small and Gandy, 2006).

**Figure 1:**
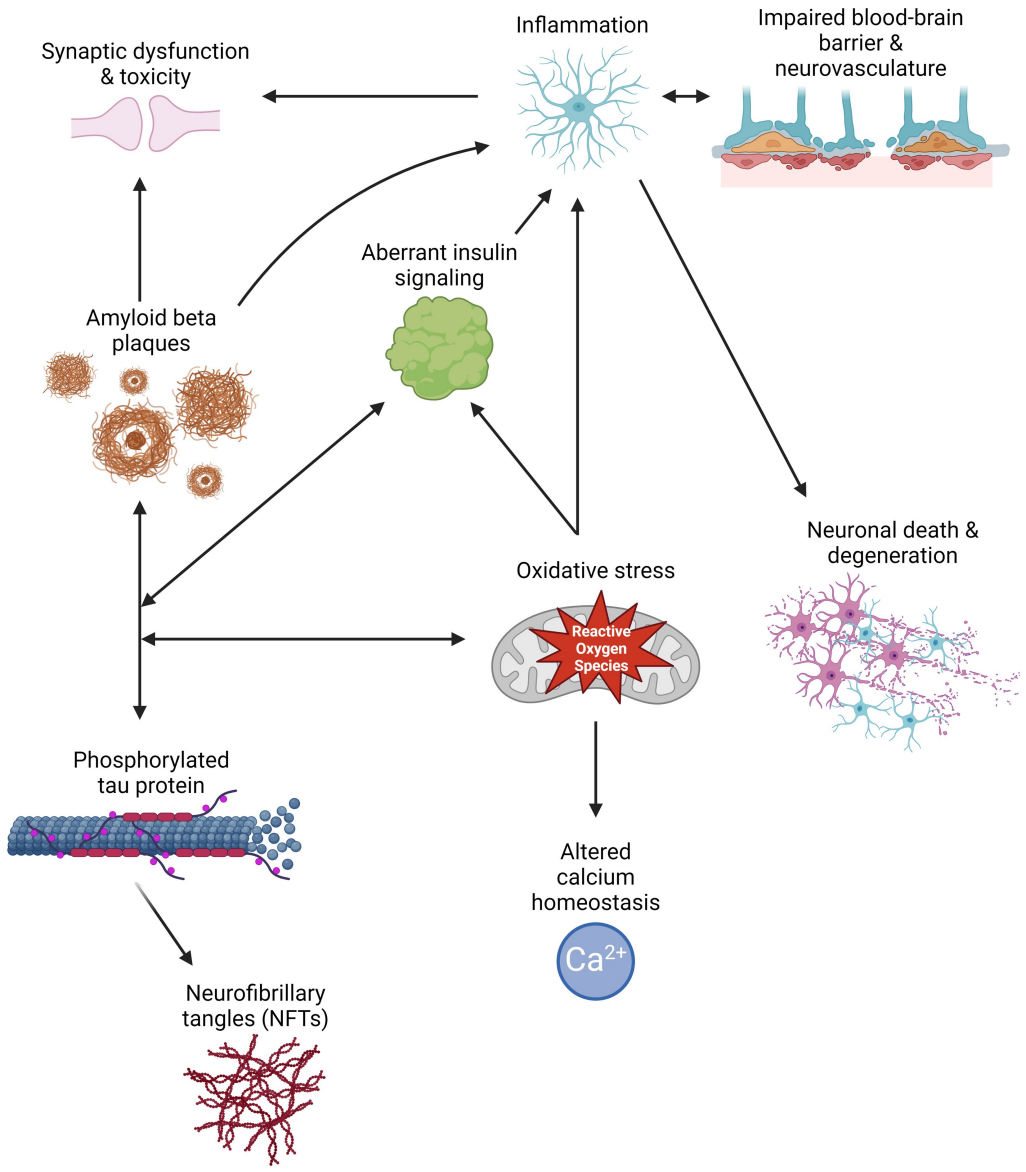
Schematic of multiple pathways that contribute to Alzheimer’s disease pathology, and how their interactions can exacerbate symptomology and progression. Created with BioRender.com.

Aβ peptides are derived from the cleavage of amyloid precursor protein (APP) by β- or γ-secretase, resulting in the generation and aggregation of insoluble extracellular plaques and soluble oligomers that interfere with neuronal signaling, morphology, and function (Li et al., 2009; Zhang H. et al., 2015; Chen et al., 2017). Intracellularly, hyperphosphorylated isoforms (p-tau) of the microtubule-associated protein tau (MAPT) accumulate in NFTs and interact with other key AD proteins such as ubiquitin and apolipoprotein E (Drummond et al., 2020). P-tau oligomers contribute to neurodegeneration by disturbing microtubule dynamics, neuronal maturation, calcium (Ca2+) homeostasis, and organelle trafficking (Alonso et al., 2006; Swanson et al., 2017). Aβ aggregation has been shown to induce p-tau redistribution from axons to cell bodies and dendrites in hippocampal rat neuronal cultures, resulting in loss of dendritic spines, mitochondria, and microtubules and an increase in Aβ plaques (Zempel et al., 2010; Chen et al., 2021). This supports the amyloid cascade theory, suggesting that Aβ aggregation precedes and exacerbates p-tau pathology in AD. However, two FDA-approved immunotherapies targeting Aβ, aducanumab and lecanemab, show modest reductions in Aβ levels and cognitive decline in some patients with mild cognitive impairment (MCI) or early AD and a 30% risk of brain swelling or bleeding (Brockmann et al., 2023). Therefore, other mechanisms must contribute to AD development and progression. Alternate theories in the field include the tau hypothesis, where p-tau drives amyloidogenic APP cleavage and is transferred between neurons in a “prion-like” manner, and the oxidative stress hypothesis that postulates that dysregulation of reactive oxygen species causes a cytotoxic cascade resulting in neurodegeneration (Tardivel et al., 2016; Roy et al., 2023). Therefore, it is essential to investigate additional mechanisms and pathways that contribute to the development and progression of AD (Figure 1) and identify the critical therapeutic window for effective treatment.

AD is a complex disorder caused by genetic alterations in key genes, with an estimated heritability of 58–79% (Gatz et al., 2006). AD is subdivided into two types based on its genetic basis: early-onset Familial AD (EOAD) and late-onset sporadic AD (LOAD) (DeTure and Dickson, 2019). EOAD is a rare autosomal dominant form of the disease affecting adults younger than 65 years old caused by mutations in the amyloid precursor protein (APP), presenilin 1 (PSEN1), and presenilin 2 (PSEN2) genes (Hsu et al., 2018). PSEN1 and PSEN2 encode the γ-secretase proteolytic subunit responsible for Aβ cleavage, with PSEN1 mutations being the most common in EOAD (Xiao et al., 2021). Conversely, LOAD is the most common form of AD in adults over 65 years of age and is highly polygenic and multifactorial (Jansen et al., 2019; Kunkle et al., 2019). Factors contributing to the development of LOAD include aging, APOE isoforms, stress, changes in the gut microbiome, and comorbidities such as cardiovascular diseases (Leszek et al., 2020; Jin et al., 2021; Nagu et al., 2021; Raulin et al., 2022). While historically, most efforts have focused on the genetic basis of AD, it is becoming increasingly appreciated that epigenetic mechanisms could play pivotal roles in AD pathogenesis, even at the preclinical stage (Kuehner et al., 2021).

Epigenetics studies stable, heritable changes in gene expression without modification of the underlying DNA sequence. Epigenetic processes usually involve chemical covalent modifications, such as methylation, in histone proteins, DNA and coding or non-coding RNA molecules, that affect gene expression at both transcriptional and post-transcriptional levels. Dynamic epigenetic regulation occurs spatially and temporally, allowing for neurogenesis and neuronal patterning during neurodevelopment and neuroplasticity throughout adulthood (Yao et al., 2016). Aberrant epigenetic alterations induced by environmental stimuli such as radiation, oxygen radicals, and the aging process result in the ectopic repression or overexpression of critical genes that contribute to disease etiology. These epigenetic alterations have been associated with the development of pathologies, including cancer, chronic neuropathic pain, and neurodegenerative diseases such as AD (Takeshima and Ushijima, 2019; Ghosh and Pan, 2022). However, the mechanisms that result in the accumulation of epigenetic modifications and how their dysregulation in the central nervous system contributes to the onset and development of AD remains largely unknown. Moreover, identifying epigenetic biomarkers for the early diagnosis and treatment of AD is essential to increase the quality of life of patients and their caregivers. Here, we systematically summarize current knowledge and emerging roles of transcriptional and post-transcriptional epigenetic mechanisms in DNA and RNA associated with AD development and progression. We also highlight their potential as biomarkers for diagnosis and treatment. This review focuses on DNA modifications including DNA cytosine methylation and hydroxymethylation, DNA adenine methylation, RNA adenine methylation, tRNA modifications, long non-coding RNAs, microRNAs, and circular RNAs. Nonetheless, other DNA and RNA modifications not described in this review have been shown to or have the potential to influence AD pathology. Other reviews have explored the roles of histone modifications in AD and other neurological disorders, such as Park et al. (2022), Santana et al. (2023), and Qin et al. (2024).

## 2 DNA modifications

### 2.1 DNA cytosine methylation (5-methylcytosine, 5mC) and hydroxymethylation (5-hydroxymethylcytosine, 5hmC)

The most predominant DNA modifications occur on the cytosine residue. 5-methylcytosine (5mC) describes the addition of a methyl group to the fifth carbon position of cytosine, usually at CpG dinucleotides, by a group of DNA methyltransferases (DNMTs) in the mammalian genome. The addition of 5mC is associated with transcriptional repression at promoters and enhancers, resulting in cell and tissue-specific gene expression (Robertson and Wolffe, 2000). In 2009, it was discovered that 5mC is oxidized into 5-hydroxymethylcytosine (5hmC) by Ten-eleven translocation (TET) enzymes, resulting in a stable and independent epigenetic mark in DNA at gene bodies and enhancers that promotes critical gene expression during development (Tahiliani et al., 2009; He et al., 2021).

DNA methylation and hydroxymethylation are enriched in the brain and dynamically regulated in neurodevelopment (Kriaucionis and Heintz, 2009; Lister et al., 2013). Its dynamics play important roles in modulating neurogenesis, synaptic transmission, learning, and memory (Hahn et al., 2013; Meadows et al., 2015; Bayraktar et al., 2020; Zocher et al., 2021). Postmortem analysis of human brain tissue of embryos, young adults, and older adults, identified a shift from 5mC to 5hmC in the 5′ flanking region of *PSEN1* associated with aging (Monti et al., 2020). Accordingly, loss of function of Dnmts or Tets result in embryonic lethality and impaired neurogenesis (Jin and Robertson, 2013; Kang et al., 2015).

Epigenetic clocks are algorithms used to estimate the age of an organism, tissue, or cells based on methylation changes at specific sites in the genome (Kabacik et al., 2022). Accelerated aging, defined as the difference between chronological and epigenetic clock age, can serve as a biomarker for functional aging and aid in age of onset predictions for neurodegenerative diseases. Several epigenetic clocks suggest that AD patients present accelerated epigenetic aging correlating with lifestyle risk factors, amyloid load, neurofibrillary tangles, and declines in memory and cognition (Levine et al., 2015; McCartney et al., 2018; Grodstein et al., 2021). A 4-year longitudinal study of blood DNA revealed that AD patients exhibit DNA methylation alterations in dementia-associated genes before their clinical diagnosis (Pérez et al., 2022). Furthermore, genome-wide analyses of postmortem human brains from the Religious Orders Study (ROS) and Memory and Aging Project (MAP) have identified whole brain and region-specific differentially methylated sites associated with known and novel AD risk loci related to cell adhesion, calcium homeostasis, and the immune system, key processes that are dysregulated in AD (De Jager et al., 2014; Semick et al., 2019). These changes are recapitulated in *in vitro* and *in vivo* animal models (Leparulo et al., 2022). AD forebrain organoids present global 5hmC alterations in known neurodevelopmental and AD risk genes (Kuehner et al., 2021). Similarly, 5xFAD mice with partial loss of Tet1 exhibit exacerbated symptom severity and aberrant 5mC and 5hmC expression at key genes, including TET3, DNMT3A, and Methyl-CpG binding protein 2 (MECP2) (Armstrong et al., 2023).

AD is caused by a combination of genetic, environmental, and lifestyle factors that impact age-related changes in the brain. Exposure to heavy metals or air pollutants, diets low in antioxidants, physical inactivity, comorbidities, and social factors can increase the risk of AD (Chen et al., 2016; Cacciottolo et al., 2017; Sinyor et al., 2020). Chronic stress and depression are known risk factors for the development of AD and accelerated aging (Wallensten et al., 2023). Mouse models of chronic stress recapitulate depression-like behaviors and exhibit global 5mC and 5hmC cortical alterations. In two separate studies, differentially hydroxymethylated regions were enriched for gene ontology terms associated with neurogenesis and synaptic signaling (Cheng et al., 2018; Kuehner et al., 2023). Notably, hypoxia-inducible factors (HIFs) Hif3 and Hif1α, known to play neuroprotective roles, were found to be associated with stress-induced methylation changes. Indeed, AD patients are reported to present lower Hif1α levels associated with neurovascular and calcium homeostasis dysfunctions that further exacerbate neuroinflammation, oxidative stress, and p-tau and Aβ depositions (Ashok et al., 2017; Mitroshina et al., 2021). DNA methylation and hydroxymethylation dynamics play important roles in AD-related pathways, and their contributions to disease onset and progression require further exploration to determine their potential as therapeutic targets.

### 2.2 DNA adenine methylation (N6-methyladenine, 6mA)

DNA N6-methyladenine (6mA) is a DNA modification on the sixth position of adenine that was thought to only be present in prokaryotes until recent technological advances enabled its identification and quantification in eukaryotes (Greer et al., 2015; Zhang G. et al., 2015; Wu et al., 2016). 6mA is thought to be placed on the DNA by N-6 adenine-specific DNA methyltransferase 1 (N6AMT1 or DAMT-1) and removed by demethylase Alkb homolog 1 histone h2a dioxygenase (ALKBH1); and appears to be enriched at exonic regions, intronic regions, and intergenic regions, as well as at retrotransposons including long interspersed nuclear elements (LINEs) (Wu et al., 2016; Yao et al., 2017; Zhang M. et al., 2020; Feng et al., 2024).

Recent studies suggest that 6mA plays a role in various stress responses. In *Caenorhabditis elegans*, 6mA is deposited at mitochondrial stress and heat stress response genes, and interacts with histone modifications to modulate the transgenerational inheritance of mitochondrial stress and hormetic heat stress adaptations (Ma C. et al., 2019; Wan et al., 2021). Similarly, 6mA has been shown to be enriched in human mitochondrial DNA (mtDNA), increases under hypoxic stress, and is associated with mtDNA gene expression repression via disruption of transcription factor A mitochondria (TFAM) binding (Hao et al., 2020). In a chronic stress mouse model, genome-wide 6mA accumulation and decreased intragenic 6mA levels negatively correlated with the upregulation of genes associated with neuronal development and function, and differentially 6mA regions overlapped with genes associated with neuropsychiatric disorders (Yao et al., 2017). Moreover, pregnant rats exposed to the brominated flame retardant α-hexabromocyclododecane gave birth to pups with motor and learning deficits that exhibited a trend toward decreased 6mA levels in the cerebellum (Fernandes et al., 2021). Similarly, neonatal rat exposure to predator odor stress was associated with altered 6mA levels in the amygdala, and sex-specific differences in 5-Hydroxytryptamine Receptor 2A (Htr2A) promoter repression and anxiety-like behavior (Kigar et al., 2017). These studies provide further evidence that 6mA plays a role in neurodevelopment. ALKBH1 knockdown mice exhibit impaired axon regeneration and the significant downregulation of 5 key neurodevelopment genes: Inhibitor of DNA Binding 1 (Id1), Ephrin A1 (Efna1), Neuritin 1 (Nrn1), Autophagy related 9B (ATG9B), and Complement C1q like 4 (C1QL4), as well as a trend toward downregulation for 14 others genes including APOE (Li et al., 2021). In accordance with these results, a preliminary study suggests that blood leukocyte 6mA levels are significantly lower in AD (Lv et al., 2022). The innate immune system plays important roles in AD through its response to pathogens, endogenous molecules such as Aβ, and debris released by necrotic cell death that contribute to pathogenic neuroinflammation (Takeuchi and Akira, 2010). The activation of murine and human cerebrospinal fluid (CSF)-derived macrophages was shown to be enhanced by the recognition of synthetic cytosolic dsDNA Gm6ATC and GTm6AC motifs, while recognition of the Cm6ATG motif attenuated macrophage activation, indicating that 6mA may modulate immune and inflammatory processes in a sequence- and context-specific manner (Balzarolo et al., 2021). Further investigation of 6mA dynamics in AD is warranted.

## 3 RNA modifications

More than 150 biochemical modifications have been identified in coding and non-coding RNAs (Boccaletto et al., 2022). This review focuses on exploring the contributions of RNA adenine methylation (N6-methyladenosine, m6A) and tRNA modifications in Alzheimer’s disease pathogenesis.

### 3.1 RNA adenine methylation (N6-methyladenosine, m6A)

RNA N6-methyladenosine (m6A) is the most abundant RNA modification, consisting of the addition of a methyl group to the sixth position of adenosine by the m6A methyltransferase complex formed by methyltransferase-like 3 (METTL3) and methyltransferase-like 14 (METTL14). m6A is removed from RNA by Fat mass obesity-associated (FTO) and alkB homolog 5 (ALKBH5) and recognized by several nuclear and cytoplasmic m6A-binding proteins, including YTHDF1/2/3 and YTHDC1/2 (Yang et al., 2018).

m6A is highly enriched in the brain, and its increased deposition is associated with adult neurogenesis, memory, learning, and BDNF and dopaminergic signaling pathways (Meyer et al., 2012; Hess et al., 2013; Li et al., 2017). Recently, m6A was also found to be dynamically regulated by extracellular signal-regulated kinase (ERK)/mitogen-activated protein kinase (MAPK) signaling. The ERK/MAPK pathway is involved in cell proliferation, differentiation, stress response, and neuroinflammation (Albert-Gascó et al., 2020; Guo et al., 2020). Activation of ERK in mouse embryonic stem cells (mESCs) increases METTL3 phosphorylation, leading to increased METTL3 deubiquination by ubiquitin specific peptidase 5 (USP5), which in turn increases METTL3 stability and m6A deposition (Sun et al., 2020). Moreover, m6A deposition on 3’UTRs is required for the localization of mRNAs, particularly calcium/calmodulin-dependent protein kinase II (CAMK2) and microtubule-associated protein 2 (MAP2), from the soma to neurites for proper synaptic and growth cone function in hippocampal neurons (Flamand and Meyer, 2022). CAMK2A is a gene known to play roles in synaptic plasticity, memory, and calcium homeostasis, and its dysregulation has been linked to AD susceptibility and pathology (Ghosh and Giese, 2015; Fang et al., 2019). MAP2 plays a key role in neurite and synapse growth and function, and its aberrant phosphorylation is associated with neuropsychiatric and neurodegenerative disorders (Grubisha et al., 2021; DeGiosio et al., 2022). Dysregulation of MAP2 is associated with tauopathies, given that MAP2 shares sequences homology with tau and can inhibit its aggregation by binding to the ends of the tau fibrils (Zhang and Dong, 2012; Holden et al., 2023).

Alterations in m6A are associated with AD pathology and progression. In C57BL/6 J mice, m6A levels decreased postnatally and increased during the aging process, resulting in tissue-specific regulation of mRNA expression and splicing. Furthermore, 5xFAD mice display decreased m6A levels in the 3’UTRs of AD-associated transcripts that correlate with decreased protein expression, despite mRNA levels remaining unchanged (Shafik et al., 2021). APP/PSEN1 mice exhibit increased m6A deposition and METTL3 activation associated with synaptic growth and function (Han et al., 2020). Conversely, AD postmortem human brains exhibit cell type-specific m6A dysregulation, with pyramidal neurons containing lower m6A and METTL3 levels associated with memory deficits, neuronal death, and neurite degeneration (Zhao et al., 2021). These results are further supported by Parkinson’s disease rats and cell model studies, where global m6A downregulation was associated with increased dopaminergic neuron death (Chen et al., 2019). Interestingly, AD glia cells exhibited increased m6A deposition, but these findings require further study (Zhao et al., 2021). METTL3 was also identified as the only m6A reader differentially activated in MCI patients (Zhao et al., 2021). In a mouse model of chronic restraint stress, physical exercise resulted in increased levels of m6A deposition by METTL3 at sites associated with synapse-related pathways in the medial prefrontal ortex, resulting in higher resilience against the effects of stress, as measured by behavioral testing. Furthermore, decreased levels of serum SAM levels in both patients with major depressive disorder (MDD) and in mice were associated with reduced resilience, suggesting that methyl donor S-adenosyl methionine (SAM) could be used as a biomarker and target for the treatment of anxiety, a risk factor for AD (Yan et al., 2022). In an Aβ-induced mouse model, loss of METTL3 in monocyte-derived macrophages decreased DNMT3A mRNA via YTHDF1 and increased macrophage recruitment, resulting in higher rates of Aβ clearance, decreased α-tubulin acetylation, and improved learning and memory scores (Yin et al., 2023). Taken together, these studies suggest that m6A plays a role in the early development and progression of AD.

### 3.2 tRNA modifications

Transfer RNAs (tRNAs) are stable RNAs involved in translation that recognize mRNA codons and provide the corresponding amino acids. tRNAs are modified post-transcriptionally as part of their maturation process, enhancing tRNA stability, translation efficiency, translation rate, and anticodon-codon specificity (Helm and Alfonzo, 2014; Agris et al., 2018). In eukaryotes, each tRNA molecule averages 13 modifications, which are most commonly found at the anticodon stem-loop at wobble position 34 and adjacent to the 3′ anticodon at position 37 (Dunin-Horkawicz et al., 2006; Jackman and Alfonzo, 2013). tRNA modifications also allow tRNAs to respond quickly to cellular stress, modulating translation to increase the production of key stress response proteins (Damon et al., 2015; Endres et al., 2015; Zhong et al., 2015). Consequently, dysregulation of tRNA modifications is associated with neurodevelopmental disorders, neurodegeneration, and brain tumorigenesis (Ishimura et al., 2014; Nagayoshi et al., 2021; Zhang et al., 2021).

Recent studies have begun to associate cytosolic and mitochondrial tRNA modifications with AD pathophysiology. The posterior cingulate cortex (PCC) is a key metabolic region affected in AD, and hypometabolism and gray matter loss in the PCC have been associated with disease progression (Scheltens et al., 2018; Lee et al., 2020). Astrocyte transcriptome analysis of human postmortem PCC samples identified 226 differentially expressed genes, including the mitochondrial-encoded gene tRNA methyltransferase 61 homolog B (*TRMT61B*) (Sekar et al., 2015). *TRMT61B* catalyzes the N1-methyladenine (m1A) modification, which is associated with tRNA folding and stability (Chujo and Suzuki, 2012; Bar-Yaacov et al., 2016). In both the 5xFAD mouse cortex and transgenic *Drosophila*, cytosolic and mitochondrial m1A hypomethylation and decreased expression of tRNA methyltransferases tRNA methyltransferase 10 homolog C (*TRM10C*), hydroxysteroid 17-beta dehydrogenase 10 *(HSD17B10)*, and tRNA methyltransferase 61 homolog A *(TRMT61A)* resulted in increased AD phenotypic severity (Shafik et al., 2022). Additionally, aberrant methylation at mitochondrial tRNA position 9 (m1A9) in the cerebellum of AD patients may also disrupt the cloverleaf structure during tRNA folding and further contribute to mitochondrial dysfunction in neurodegeneration (Silzer et al., 2020; Xiong et al., 2023).

Recently, several tRNA modifying enzymes affecting the wobble position were found to be differentially expressed throughout the human AD brain. Among these were NOP2/Sun RNA methyltransferase 2 (*NSUN2*), adenosine deaminase tRNA specific 2 and 3 (*ADAT2/3*), alkB homolog 8 *(ALKBH8)*, and the acetyltransferase elongator complex subunits 1, 3, and 6 (*ELP1/3/6*). The elongator complex has been reported to play roles in neurodevelopment and neurological disorders, such as amyotrophic lateral sclerosis (ALS), intellectual developmental disorders, neuropathies, and dysautonomias (Simpson et al., 2009; Cohen et al., 2015; Bento-Abreu et al., 2018; Gaik et al., 2022). Notably, *ELP3* expression decreases in response to aggregated Aβ across several AD models, resulting in hypomodifications that contribute to tRNA instability and impaired translation (Pereira et al., 2024). Another tRNA modifying enzyme that may contribute to AD pathogenesis and progression is cytoplasmic cysteinyl-tRNA synthetase (CARS). In APP/PS1 mice and SH-SY5Y neuronal cells, increased CARS expression correlated with AD severity and localized to Aβ plaques, exacerbating memory deficits and inflammatory microglial activation (Qi et al., 2024). Ongoing studies into the functions of tRNA modifications and their modifying enzymes will increase our understanding of their role in AD pathology, neurodegeneration, and age-related diseases.

## 4 Other regulatory RNAs

**Figure 2:**
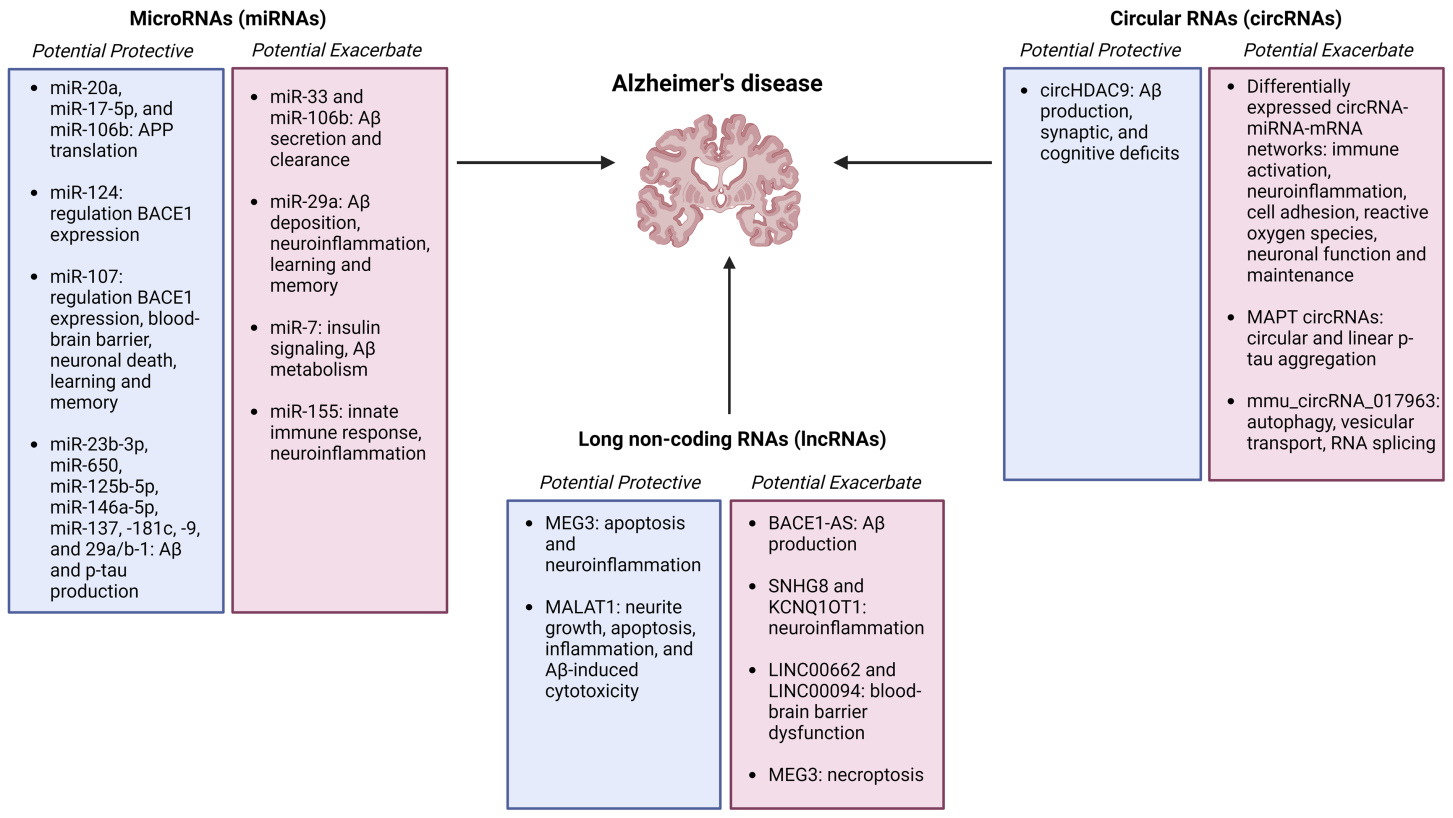
Summary figure of non-coding RNAs (microRNAs, circular RNAs, and long non-coding RNAs) identified as potentially protective or exacerbating in Alzheimer’s disease onset, pathology, and progression Created with BioRender.com.

Regulatory RNAs constitute a wide range of non-coding RNAs that interact with DNA, RNA, and proteins in numerous cellular pathways, and may serve as biomarkers and therapeutic targets for Alzheimer’s disease (Figure 2).

### 4.1 Long non-coding RNAs

Long noncoding RNAs (lncRNAs) are over 200 nucleotides long and do not encode a protein product. lncRNAs regulate gene and protein expression in both *cis* and *trans* by forming complexes with DNA, RNA, and RNA binding proteins (RBPs) in tissue-specific and ubiquitous manners (Lee, 2012; Jiang et al., 2016). lncRNAs play transcriptional roles in chromatin architecture and RNA polymerase II activity, post-transcriptional roles in splicing and translation, and post-translational roles as protein scaffolds (Romero-Barrios et al., 2018; Hou et al., 2019; Karakas and Ozpolat, 2021; Johnsson et al., 2022). Approximately 40% of currently identified lncRNAs are specifically expressed in the brain, and many have been implicated in neuropathological processes via annotation of the GENCODE human gene catalog and experimental validation in rodent models, including synaptic plasticity, neuroinflammation, blood–brain barrier (BBB) permeability, and neuropathic pain (Derrien et al., 2012; Zhao et al., 2013).

β-secretase 1 (BACE1) is a key enzyme in the cleavage of APP into amyloidogenic Aβ. BACE1 expression is modulated by the lncRNA BACE1-AS derived from the antisense transcript. In the presence of Aβ plaques, BACE1-AS is upregulated and increases BACE1 protein expression by functioning as a competing endogenous RNA with miRNAs known to regulate BACE1. This creates a positive feedback loop that further drives Aβ production and AD pathology that has been validated *in vitro* and *in vivo* across several human and mouse tissues and cell lines (Faghihi et al., 2008; Zeng et al., 2019).

One critical lncRNA involved in neuroinflammation and BBB permeability is small nucleolar RNA host gene 8 (SNHG8). In ischemic stroke models, SNHG8 contributes to neuronal apoptosis, microglial activation, and neuroinflammation by sponging miR-449c-5p and miR-425-5p, resulting in the downstream regulation of sirtuin 1 (SIRT1) (Tian et al., 2021; Zhang et al., 2022). SIRT1 is a Nicotinamide adenine dinucleotide (NAD+)-dependent deacetylase enzyme involved in a wide range of AD-associated pathways, including Aβ and tau metabolism, neuronal development, memory, and inflammation (Min et al., 2010; Wang et al., 2013; Corpas et al., 2017). Moreover, in human AD, mouse, and *in vitro* tauopathy models expressing mutant *MAPT*, increased p-tau levels resulted in decreased SNHG8 expression, increased stress granule formation, and elevated cytotoxic granule-associated RNA binding protein TIA1 (Bhagat et al., 2023). Other dysregulated lncRNAs contributing to BBB permeability in AD via interactions with RBPs include LINC00662 and LINC00094 (Zhu et al., 2019; Liu et al., 2020). Additionally, KCNQ1OT1 has been shown to increase neuroinflammation and neuronal death in microglial HMC3 cells by sponging miR-30e-3p, resulting in increased expression of the NLR family pyrin domain containing 3 (NLRP3) inflammasome (Song et al., 2021).

Neuronal death is a key pathological hallmark of AD. The lncRNA maternally expressed 3 (MEG3) is upregulated in AD patients, and a human xenograft model suggests that MEG3 expression is sufficient to induce necroptosis (Balusu et al., 2023). Programmed inflammatory cell death is hypothesized to exacerbate AD progression by promoting microglial activation, synaptic loss, and BBB dysfunction (Zhang et al., 2023). Conversely, an AD Aβ_25-35_ microinjection rat model study suggests that MEG3 overexpression exerts a neuroprotective role by inhibiting apoptosis and inflammation through a MEG3-miR-93-Proteinase kinase B (PI3K/AKT) pathway (Yi et al., 2019). Metastasis associated lung adenocarcinoma transcript 1 (MALAT1) confers a neuroprotective role in AD by promoting neurite outgrowth and inhibiting apoptosis, inflammation, and Aβ_1-42_ induced cytotoxicity by interacting with miR-125, -200a, -26a, -26b, and their downstream targets Prostaglandin-endoperoxide Synthase 2 (PTGS2), Cyclin-dependent Kinase 5 (CDK5), Forkhead Box Q1 (FOXQ1), and Receptor Tyrosine Kinase (RTK) EphA2 (Ma P. et al., 2019). Further investigation into the roles of MEG3 and MALAT3 in AD pathology as possible therapeutic targets is warranted.

### 4.2 MicroRNAs

MicroRNAs (miRNAs) are single-stranded non-coding RNAs approximately 22 nucleotides long. miRNAs are transcribed from DNA into primary miRNAs (pri-miRNAs) and further processed into precursor miRNAs (pre-miRNAs) and mature miRNAs by DROSHA and Dicer complexes, respectively. miRNAs can regulate gene expression via translational repression or mRNA degradation and have been implicated in AD pathogenesis (O’Brien et al., 2018).

Aβ production and stability are under the regulation of multiple miRNAs. In human HeLa cells, miR-20a, miR-17-5p, and miR-106b modulate APP translation by binding to the 3’UTR without affecting mRNA levels. Despite all three miRNAs exhibiting significantly decreased levels during mouse embryonic stem cell development and differentiation, miR-106b showed the most significant decrease in human AD brains (Hébert et al., 2009). Additionally, overexpression of miR-106b in both mouse neuroblastoma N2A and human hepatocyte HepG2 cells promotes Aβ secretion and impairs its clearance by targeting the 3’UTR of the ATP-binding cassette transporter A1 (ABCA1) and suppressing its expression (Kim et al., 2012). ABCA1 is required to maintain APOE levels and lipidation, and its dysregulation has been implicated in AD and other inflammatory diseases (Wahrle et al., 2004; Lewandowski et al., 2022). In 5xFAD mice, miR-29a loss of function improved AD-associated deficits in learning and memory, Aβ deposition, and neuroinflammatory markers such as activated astrocytes and microglia. The predicted downstream targets of miR-29a, plexin-A1 (Plxna1), and WD Repeat and FYVE Domain Containing 1 (Wdfy1) have been associated with neuroinflammation, axon guidance, and neurogenesis in mice and rats (Mei et al., 2023).

Insulin is essential for glutamatergic signaling, BBB integrity, vasodilation, neuronal proliferation, differentiation, cognition, and memory (Åberg et al., 2003; Hallschmid et al., 2007; Sawallisch et al., 2009; Heni et al., 2011; Irvine et al., 2011; Kuwabara et al., 2011; Chirivella et al., 2017; Pomytkin et al., 2019; Fetterly et al., 2021; Wang et al., 2022; Nguyen et al., 2023). Changes to insulin signaling, particularly desensitization to insulin, result in chronic inflammation and are a known risk factor for AD across human and rodent *in vitro* and *in vivo* models (Baker et al., 2011; Blázquez et al., 2014; Craft et al., 2017). miR-7 is elevated in the cortex of AD patients and regulates critical insulin and Aβ metabolic genes, including ABCA1, insulin receptor (INSR), insulin receptor substrate 2 (IRS-2), insulin-degrading enzyme (IDE), and liver x receptor (LXR) (Fernández-de Frutos et al., 2019). Furthermore, miR-7 has been shown to play roles in neuroinflammatory pathways in astrocytes and mice (Dong et al., 2016; Yue et al., 2020). Another pro-inflammatory miRNA known to modulate the innate immune response is miR-155 (Hu et al., 2022). In 12-month 3xTg AD mice, upregulation of miR-155 correlated with increased neuroinflammatory markers and intracellular Aβ accumulation. This could create a positive feedback loop, as Aβ can activate the c-Jun N-terminal Kinase (JNK) signaling pathway, resulting in additional miR-155 overexpression and downregulation of its target suppressor of cytokine signaling 1 (SOCS-1) (Guedes et al., 2014).

miRNAs can also play protective roles in AD. miR-124 and miR-107 levels are significantly decreased in AD, resulting in the upregulation of BACE1 mRNA levels (Wang et al., 2008; An et al., 2018). In human brain microvascular endothelial cell (EC) co-cultures modeling the BBB, miR-107 overexpression stimulated tight junction-related proteins to repair BBB permeability (Liu et al., 2016). Additionally, miR-107 mimic administration in C57BL/6J mice alleviated behavioral and pathological symptoms of AD, including pyramidal neuronal loss and deficits in spatial memory and hippocampal long-term potentiation (Shu et al., 2018). Similarly, reductions of miR-23b-3p levels in APP/PSEN1 mice resulted in increased p-tau and neuronal death via impaired inhibition of its target glycogen synthase kinase-3β (GSK-3β). Other miRNAs downregulated in early AD that may inhibit Aβ and p-tau production include miR-23b-3p, miR-125b-5p, miR-146a-5p, miR-137, miR-181c, miR-9, and miR-29a/b-1 (Geekiyanage and Chan, 2011; Jiang et al., 2022; Yashooa and Nabi, 2022). For instance, targeting elevated miR-29a levels in 5xFAD mice with an AAV miR-29a sponge results in decreased miR-29a expression, Aβ deposition, and expression of neuroinflammatory markers, as well as improvements in learning and memory (Mei et al., 2023). Another potential therapeutic target for AD is miR-650, which has been found to be significantly increased in the human AD cortex and targets the key AD genes *APOE, PSEN1,* and *CDK5.* Overexpression of miR-650 in APP/PSEN1 mice results in the decreased levels of *CDK5* and the downstream inhibition of Aβ and neuronal loss (Lin et al., 2023). Further investigations into the roles of miRNAs as protective or exacerbating factors in AD will aid in the understanding of the disease.

### 4.3 Circular RNAs

Circular RNAs (circRNAs) are single-stranded, covalently closed loops formed by non-canonical pre-mRNA back splicing (Salzman et al., 2012). They are stable, conserved, and widely expressed in neuronal tissues (Jeck et al., 2013; Ashwal-Fluss et al., 2014). CircRNAs most commonly function as miRNA sponges, where a circRNA binds in a sequence-specific manner to a miRNA or miRNA family, preventing its binding to downstream target genes and proteins (Hansen et al., 2013). CircRNAs accumulate with age in the brain depending on the host gene expression in cell type- and region-specific manners (Gruner et al., 2016; Chen et al., 2018). In AD patient samples from the Knight Alzheimer Disease Research Center (ADRC) and the Mount Sinai Brain Bank (MSBB), circRNA expression precluded AD symptoms, correlated with diagnosis and symptom severity, and associated with genes and miRNAs involved in AD-related pathways across several brain regions (Dube et al., 2019; Lo et al., 2020). Per these findings, the MAPT locus can generate two circRNAs, exon 12 → 7 and 12 → 10, resulting in the continuous rolling circle translation of tau proteins due to the lack of a stop codon and adenine deaminase acting on RNA (ADAR) activation, promoting circular and linear tau protein aggregation (Welden et al., 2022).

AD mouse models recapitulate the circRNA dysregulation implicated in AD postmortem patient samples. Senescence Accelerated Mouse-Prone 8 (SAMP8) mice are utilized to study age-related cognitive decline and recapitulate cognitive and behavioral AD phenotypes. SAMP8 8- and 10-month mice exhibit hippocampal circRNA dysregulation that increases with age and may be associated with AD progression and severity. One significantly dysregulated circRNA, mmu_circRNA_017963, was predicted to sponge mmu_miR_7033-3p and affect downstream AD signaling pathways, including autophagy, vesicular transport, and RNA splicing (Huang et al., 2018). In APP/PSEN1 mice, circHDAC9 sponges aberrantly elevated miR-138, resulting in increased levels of its target SIRT1 and amelioration of Aβ production, synaptic, and cognitive deficits (Lu et al., 2019). Furthermore, Tg2576 and APP/PSEN1 mice exhibit differentially expressed circRNA-miRNA-mRNA networks associated with AD pathology, including upregulation of immune and inflammatory activation, cellular adhesion, and reactive oxygen species; and downregulation of synapse and dendrite function and maintenance (Lee et al., 2019; Ma N. et al., 2019).

## 5 Discussion

Epigenetic alterations of DNA and RNA methylation, acetylation, and the many non-coding RNA species provide another dimension to the investigation of the pathological mechanisms underlying AD onset and progression.

Not discussed in this review are the numerous post-translational modifications of histone proteins that alter chromatin accessibility and protein interactions, including methylation, acetylation, phosphorylation, ubiquitination, and SUMOnylation among others. Briefly, histone modifications and histone-modifying enzymes that catalyze their addition or removal, such as histone acetylatylases (HATs) and histone deacetylases (HDACs), have been implicated in AD-related processes such as impaired neurogenesis, memory deficits, and decreased neuronal survival (Park et al., 2022; Santana et al., 2023). Recent developments using HDAC inhibitors to treat cancers, ALS, and other neurological diseases show promising results in ameliorating symptoms and highlight the necessity of exploring these new approaches to treat AD (Li et al., 2020; Bondarev et al., 2021).

This review focused on the emerging roles of RNA adenine methylation (m6A), tRNA modifications, lncRNAs, miRNAs, and circRNAs. However, several other RNA modifications and species have been implicated in age-related diseases, including AD. A meta-analysis of human brain datasets identified RNA editing events in transcripts for several protein-coding genes associated with AD, including synaptotagmin 11 (SYT11) and ORAI calcium release-activated calcium modulator 2 (ORAI2) (Ma et al., 2021). Synaptotagmins interact with APP to promote Aβ production, and ORAI2 is involved in calcium homeostasis. ORAI2 overexpression has been associated with dysregulated extracellular store-operated calcium entry (SOCE), synaptic plasticity, neuronal spine morphology, and increased Aβ42 accumulation (Gautam et al., 2015; Huang et al., 2020; Scremin et al., 2020). Recently, altered small RNA modification profiles have also been associated with AD, offering a potential avenue of investigation (Zhang X. et al., 2020). An additional RNA species emerging as a potential biomarker for AD are small non-coding tRNA fragments (tRFs). tRFs are generated from precursor and mature tRNA constitutively or in response to cellular stress (Yamasaki et al., 2009; Elkordy et al., 2018). A recent study of human AD samples found increased levels of tRFs in the hippocampus, including two Trf5-ProAGG isoforms that warrant further study to determine their possible correlation with aging and AD severity (Wu et al., 2021).

Another epigenetic mechanism associated with AD that needs further study is transposable elements (TE). TEs are non-coding regulatory DNA sequences that move their position in the genome via “copy-paste” mechanisms as retrotransposons that “cut-paste” in the genome as DNA transposons. TEs are associated with aging, cellular senescence, and neurodegenerative diseases. Phosphorylated tau levels elevate TE transcription in human and *D. melanogaster* brains, resulting in increased neuroinflammation via retrotransposon-derived double-stranded RNAs (dsRNAs) (Guo et al., 2018; Ochoa et al., 2023). Additionally, the silencing of PIWI-interacting RNAs (piRNAs) leads to the transcription of silenced TEs, and contributes to their dysregulation in LOAD and tauopathies (Sun et al., 2018; Macciardi et al., 2022). Future studies should continue exploring the role of TEs-associated mechanisms in aging and neurodegeneration.

## Author contributions

PM-F: Writing – original draft, Writing – review & editing. PJ: Writing – review & editing. BY: Writing – review & editing.
